# Omental incarceration secondary to uterine perforation after dilatation curettage: Conservative approach in sub‐saharan Africa

**DOI:** 10.1002/ccr3.9480

**Published:** 2024-10-17

**Authors:** Ozer Birge, Ilkan Kayar, Fatthy Bello

**Affiliations:** ^1^ Maternité de l'Amitié Turqui‐Niger Hospital Department of Obstetric and Gynecology Niamey Niger; ^2^ Osmaniye State Hospital Department of Obstetric and Gynecology Osmaniye Turkey

**Keywords:** conservative approach, dilatation curettage, omental incarceration, uterine perforation

## Abstract

**Key Clinical Message:**

Complications of uterine perforation and herniation of intra‐abdominal organs into the perforated area may occur after gynecological operations. They can be treated with a conservative approach, especially in cases whose vital signs are stable, laboratory values are within normal limits and their general condition is suitable. We aim to present the conservative treatment without using surgical and medical methods as it is the first case in the literature.

**Abstract:**

Uterine perforation is a rare but potentially serious complication of curettage for gynecologic or obstetric reasons especially during and after operative procedures and the application of intrauterine contraceptive devices. The incidence of uterine perforation during gynecological operations is between 0.002–1.7. A 30‐year‐old multiparous case with a 7‐week 4‐day pregnancy was diagnosed with incomplete abortion at an external center due to vaginal bleeding. The case underwent suction curettage. After 24 h, revision suction curettage was performed due to the finding of rest in the endometrium during the ultrasonographic control, and the case was diagnosed with uterine perforation and omental herniation due to this. We highlighted the importance of a thorough gynecological assessment following a dilated suction curettage (D&C) procedure that includes a careful clinical examination and a detailed ultrasound evaluation.

## 1 INTRODUCTION

Although the incidence of uterine perforation is between 0.002–1.7, it has been reported that, especially recently, there has been an increase in the percentage, and diagnosis of uterine perforation after endoscopic procedures, which are minimally invasive, and for which operative methods are frequently used, and its incidence is about 1.6%.^1^, ^2^ Uterine perforation is a rare but potentially serious complication of curettage for gynecologic or obstetric reasons, especially during, and after operative procedures. Uterine perforation is a more common complication, particularly in the following situations that increase the risk of uterine perforation: uterine anomalies, infections, termination of pregnancy at young ages and late ages, retrovert uterine structure, not giving vaginal birth, previous uterine cervical surgeries, current pregnancy status, and postmenopausal period^3^ Pregnancy and evacuation of pregnancy material are the most common cases where uterine perforation is observed.^3^

In the complications that develop during the evacuation of pregnancy products by suction curettage, it is observed that abdominal organs such as the omentum, small intestines, colon, and colon epiploicae are brought to the perforated part of the uterus spontaneously or iatrogenically during the procedure.^4^, ^5^, ^6^

In this case, the patient underwent revision curettage twice 24 h apart due to incomplete abortion and then was evaluated in the obstetric emergency department due to abdominal pain and was diagnosed with omental herniation secondary to uterine perforation. We aim to present the conservative treatment without using surgical and medical methods as it is the first case in the literature.

## 2 CASE HISTORY/EXAMINATION

30‐year‐old patient has a history of five pregnancies and four normal deliveries, and her last pregnancy lasted 7 weeks and 4 days. She was diagnosed with incomplete abortion in an outpatient hospital due to vaginal bleeding and she underwent a revision curettage. Due to observing irregularity and a thickness of 26 mm in the endometrium on ultrasound control 24 h later, the second revision curettage was performed. Three days after discharge, the patient was evaluated in the obstetrics and gynecology emergency department due to lower abdominal pain. Vaginal bleeding was within normal limits; gas and stool output was normal; spontaneous micturition was normal, abdominal examination revealed tenderness, especially in the lower quadrants, and the hemoglobin value in the blood tests was 10.2 gr/dL. Our case had no comorbid additional disease. Blood pressure, pulse and fever measurements were within normal values.

## 3 METHODS (DIFFERENTIAL DIAGNOSIS, INVESTIGATIONS, AND TREATMENT)

**FIGURE 1:**
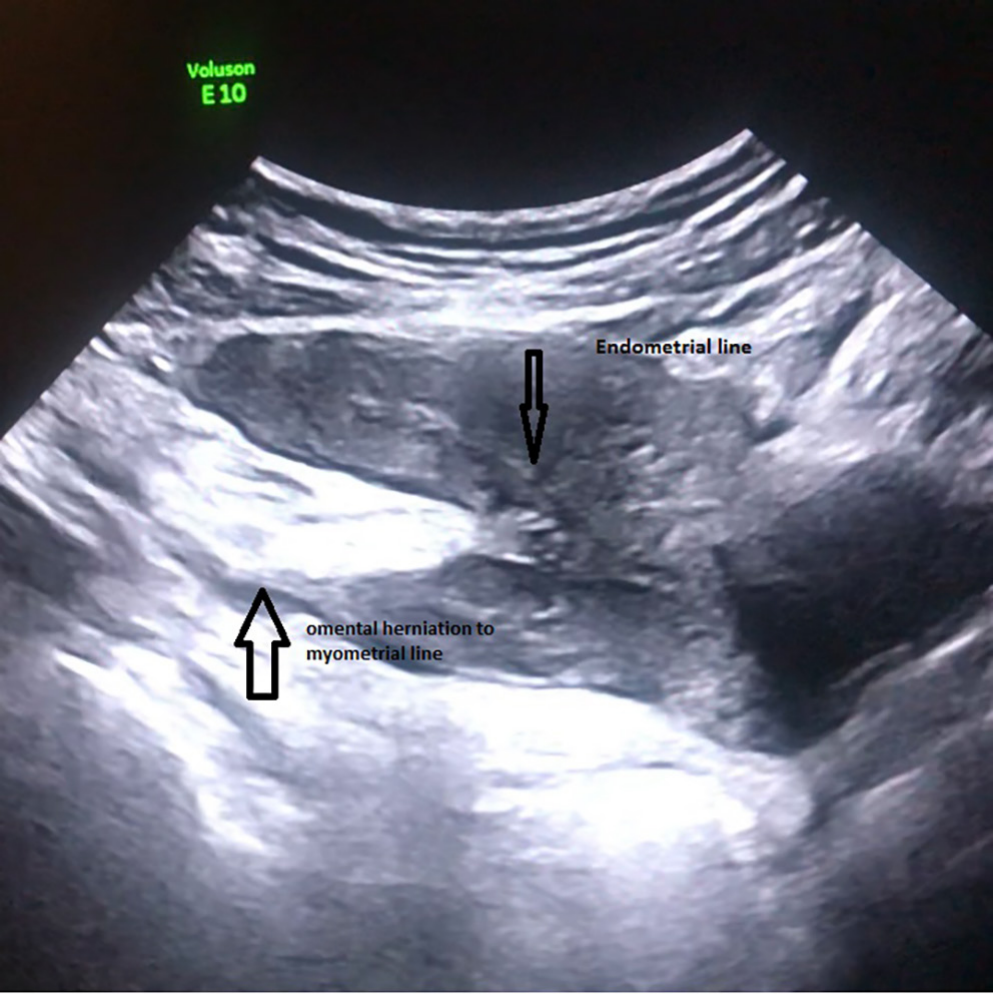
Transabdominal ultrasonographic image of the patient before the operation.

**FIGURE 2:**
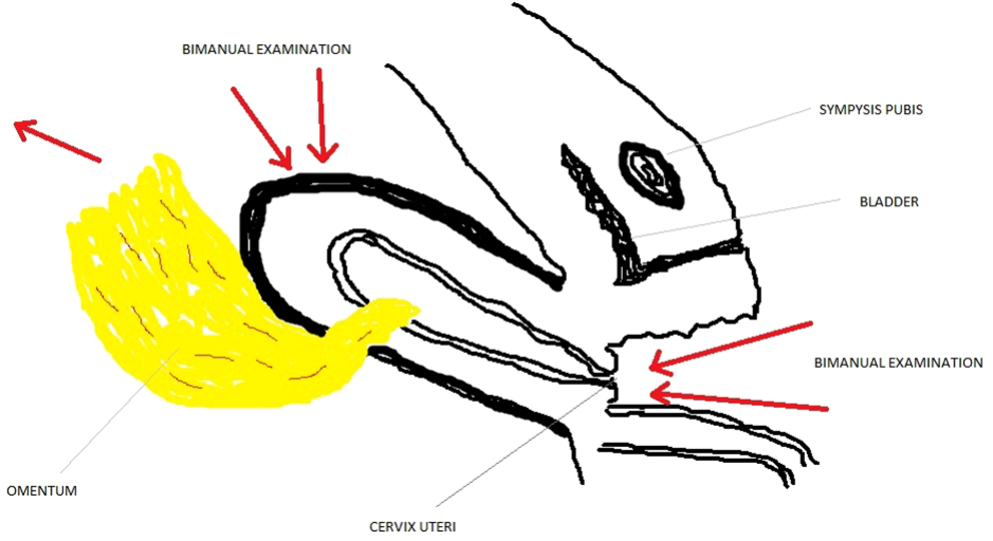
Illustrative image of bimanual vaginal examination as conservative treatment.

**FIGURE 3:**
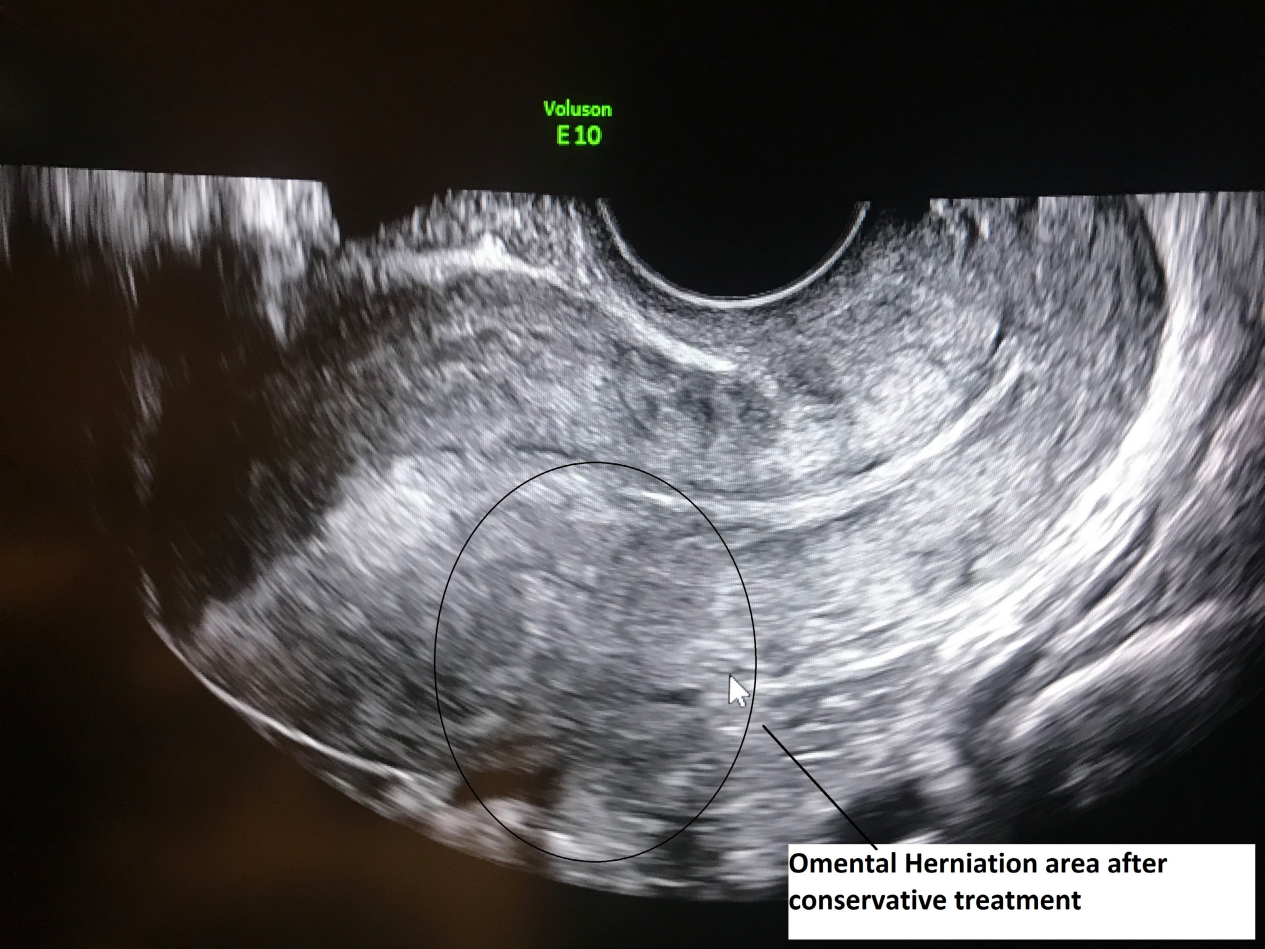
Transvaginal ultrasonographic image of the case after conservative approach.

According to the transabdominal ultrasonography performed on the gynecology table, the lesion with calcific continuity extending from the junction of the corpus isthmus at the uterus posterior to the superior pelvis was evaluated as omental or intestinal epiploicae (Figure 1). In the abdominal examination of the patient with lower abdominal pain, no rebound or defense findings were observed. It was observed that the widespread sensitivity increased from under the umbilicus to the pubic area. In the speculum vaginal examination; it was observed that the bleeding continued in the form of spots. No foul‐smelling vaginal discharge was observed. It was observed that the cervix uteri collum movements were painful. It was learned that spontaneous micturition was normal. It was learned that gas output was normal, but there was a complaint of constipation. Detailed physical examination and ultrasonographic evaluation of the patient and her relatives who were diagnosed with omental herniation after uterine perforation were given detailed information and their consents were obtained. Then, considering that there was no widespread adhesion because it was a recently developed condition, a bimanual examination was performed on the patient who first took the lithotomy position, and a gentle massage was given especially to the uterus posterior to ensure widespread movement of the uterus superiorly and anteriorly. Then, the patient, who felt pain before, relieved afterward, and taken under observation in the ward (Figure 2). One week later, the follow‐up ultrasonography showed normal anatomy of the uterus and pelvic organs (Figure 3).

## 4 CONCLUSION AND RESULTS

All intrauterine procedures should be performed with caution, and ultrasound guidance should be considered according to the circumstances. Although most uterine perforations are spontaneously resolved, they still represent one of the most severe complications and a source of long‐term complications, especially when abdominal viscera is involved.

## 5 DISCUSSION

Although cervical dilatation and uterine suction curettage/evacuation, and particularly operative hysteroscopic procedures, which are the most common minor surgical procedures in gynecology and obstetrics, are considered safe and simple, they might lead to very serious complications such as uterine perforation, especially during gynecologic, and obstetric training. Although the actual incidence is not clearly known, it is reported to be very rare because they might heal without diagnosis even if perforation occurs in some cases. In the literature, Amarin et al. reported a uterine perforation rate of 0.2% in a study evaluating about 12,000 minor surgical interventions.^7^

It should always be noted that uterine perforation might develop in gynecological and obstetric interventions. The most important point for the clinician is to recognize the development of uterine perforation and not continue with the procedure. In order to minimize perforation and similar complications, especially in procedures performed under anesthesia, a bimanual gynecological examination should be performed after a good clinical examination, the cervix uterine should be thoroughly evaluated, and if necessary, cervical ripening drugs should be used, and the procedure should be performed under ultrasound guidance.^7^, ^8^

If uterine perforation develops despite all precautions and the clinician fails to recognize this and continues with the procedure, the risk of injury to neighboring pelvic organs increases. After uterine perforation, especially intestinal injuries, omentum, colon epiploicae, intra‐abdominal solid organ injuries, and even great vessel injuries with high morbidity and even mortality were reported in the literature.^9^ This patient underwent revision curettage twice 24 h apart due to incomplete abortion after an early pregnancy loss, and she presented with abdominal pain again in the early period. The patient with stable clinical findings did not undergo any surgical and/or medical treatment but a bimanual examination was performed in the lithotomy position under outpatient clinic conditions. The uterus was lifted towards the anterior area and the omental piece located in the perforated area was spontaneously separated. In the literature, laparoscopic and/or hysteroscopic methods and even laparotomic methods were used in some cases.^4^, ^5^

In a review article published in 2024; It has been stated that the hysteroscopic mini resectoscope can be used easily, especially during diagnostic hysteroscopy, and thus can enable the detection and diagnosis of intrauterine pathologies and their treatments with less complication rates.^10^

In this region with limited resources, we think that it is appropriate to use conservative treatment methods such as waiting, and observation before a second curettage, even if there is light bleeding or for irregular endometrium.

However, in patients who are clinically stable and present early after perforation, a bimanual examination on the gynecology table, and mobilization of the uterus, and orientation of the intestinal structures to the upper abdomen in the lithotomy position should be recommended especially in underdeveloped societies with lack of required facilities.

The strengths of this study are that patients can be diagnosed and treated with conservative methods in Sub‐Saharan Africa, a region with limited resources. However, the fact that it is a single case presentation and the lack of endoscopic methods such as hysteroscopy shows our limitations.

Complications of uterine perforation and herniation of intra‐abdominal organs into the perforated area may occur after gynecological operations. They can be treated with a conservative approach, especially in cases whose vital signs are stable, laboratory values are within normal limits and their general condition is suitable.

We highlighted the importance of a thorough gynecological assessment following a D&C (dilated suction curettage) procedure that includes a careful clinical examination and a detailed ultrasound evaluation. Further studies with more cases are needed to show that the conservative approach is more appropriate in poor regions with limited resources.

## AUTHOR CONTRIBUTIONS

**Ozer Birge:** Resources; software; supervision; validation; visualization; writing – original draft; writing – review and editing. **Ilkan Kayar:** Software; supervision; visualization; writing – original draft. **Fatthy Bello:** Resources; supervision; visualization; writing – review and editing.

## FUNDING INFORMATION

Not applicable.

## CONFLICT OF INTEREST STATEMENT

All authors have no conflicts of interests to declare.

## ETHICS STATEMENT

Informed consent was obtained from the patients. Our institution does not require ethical approval for case reports.

## CONSENT

Written consent for publication was obtained from the patient.

## Data Availability

The data that supports the findings in this study is available from the corresponding author upon reasonable request.
